# Soil conditioner improves soil properties, regulates microbial communities, and increases yield and quality of *Uncaria rhynchophylla*

**DOI:** 10.1038/s41598-024-64362-4

**Published:** 2024-06-11

**Authors:** Qian Liu, Honghao Cui, Wansheng Yang, Fang Wang, Heng Liao, Qing Zhu, Song Qin, Ping Lu

**Affiliations:** 1 Center for R&D of Fine Chemicals, Guizhou University, Guiyang, 550025 China; 2 Institute of Soil Fertilizer, Guizhou Academy of Agricultural Sciences, Guiyang, 550006 China; 3 Guizhou Industry Polytechnic College, Guiyang, 550008 China

**Keywords:** *Uncaria rhynchophylla*, Soil conditioners, Microbial communities, Yield, Quality, Biochemistry, Microbiology, Plant sciences

## Abstract

*Uncaria rhynchophylla* is an important traditional herbal medicine in China, and the yield and quality of *Uncaria rhynchophylla* can be improved by suitable soil conditioners because of changing the soil properties. In this paper, *Uncaria rhynchophylla* associated alkaloids and soil microbial  communities were investigated. The field experiment was set up with the following control group: (M1, no soil conditioner) and different soil conditioner treatment groups (M2, biomass ash; M3, water retention agent; M4, biochar; M5, lime powder and M6, malic acid). The results showed that M2 significantly increased the fresh and dry weight and the contents of isorhynchophylline, corynoxeine, isocorynoxeine, and total alkaloids. *Acidobacteria, Proteobacteria, Actinobacteria*, and *Chloroflexi* were major bacterial phyla. Correlation analysis showed that fresh and dry weight was significantly positively correlated with *Acidobacteria*, while alkali-hydrolyzable nitrogen, phosphatase activity, fresh and dry weight, corynoxeine, and isocorynoxeine were significantly negatively correlated with *Chloroflexi*. The application of soil conditioner M2 increased the abundance of *Acidobacteria* and decreased the abundance of *Chloroflexi*, which contributed to improving the soil nutrient content, yield, and quality of *Uncaria rhynchophylla*. In summary, biomass ash may be a better choice of soil conditioner in *Uncaria rhynchophylla* growing areas.

## Introduction

*Uncaria rhynchophylla* is an important herbal medicine and its essential constituent alkaloids are often used to treat convulsions, hypertension, epilepsy, eclampsia, and cerebral disorders^1^. *Uncaria rhynchophylla* is mainly planted on mountain slopes, and its cultivation area is gradually increasing as its economic value grows. However, due to the limitations of topography and geomorphology, as well as problems such as improper fertilization and mismanagement during the cultivation of *Uncaria rhynchophylla*, soil nutrient imbalance, and microbial community structure have received damage, which ultimately leads to lower yields of *Uncaria rhynchophylla* and negative impacts on the local ecological environment^2^. Therefore, the improvement of soil conditions has become an urgent problem in the cultivation of *Uncaria rhynchophylla*.

With the development of industrial technology, the application of soil conditioners has gradually developed into a modern method of soil improvement^3^. It has been demonstrated that the incorporation of soil conditioners into the soil can improve the soil properties and increase its productivity^4^. In terms of functionality, they not only compensate for the limitations of conventional organic fertilizers but also further extend and intensify the fertility of the soil through the introduction of specific organic constituents^5^. In traditional fertilization practices, inappropriate application of fertilizer and a single type of fertilizer are key factors that lead to soil acidification, reduce soil fertility, and affect crop yields^6^. Compared to traditional fertilizer application methods, soil conditioner application helps to alleviate soil acidity^7^, prevent soil erosion^8^, reduce root bioavailability, and minimize the uptake of harmful soil substances^9^, thereby improving plant survival^8^. Furthermore, soil conditioners can enhance the nutrient content of soil^10^, and increase the activity of soil enzymes such as urease, sucrase, and catalase^2^, thereby contributing to improved soil environment^11^ and increased crop yields^12^. It is noteworthy that some studies have indicated that the application of soil conditioners may result in a reduction in microbial diversity^11,13^. The impact of soil amendments on the diversity and structure of soil microbial communities remains to be elucidated. However, most of the current research concentrates on the response of soils to single types of organic fertilizers or soil amendments. Consequently, further in-depth research is required to explore the potential synergistic effects of combining these approaches^14^.

In response to the current problem of reduced soil fertility and *Uncaria rhynchophylla* yield due to improper fertilizer application during *Uncaria rhynchophylla* cultivation, this study concluded that it is crucial to improve *Uncaria rhynchophylla* yield and quality by selecting suitable soil conditioners to achieve green and efficient development. Therefore, a series of field experiments were conducted to investigate the impact of five different soil conditioners on the yield and quality of *Uncaria rhynchophylla*. The main research objectives were to (1) ascertain the efficacy of various soil conditioners in enhancing the yield and quality of *Uncaria rhynchophylla*; (2) elucidate the impact of diverse soil conditioners on the physicochemical properties of the soil; (3) reveal the impact of diverse soil conditioners on alterations in soil bacterial microbial community diversity and community structure, and soil enzyme activity, and (4) explore the influence of improvements in soil properties, changes in the soil microbial community, and elevated enzyme activity on the yield and quality of *Uncaria rhynchophylla*. Ultimately, the most suitable soil conditioners were identified, providing a scientific theoretical basis for improving soil fertility and crop yield during the actual production of *Uncaria rhynchophylla*.

## Materials and methods

### Experimental site and design

The experiment was conducted in Jianhe County, Guizhou Province, China, from 2020 to 2021, with geographical coordinates at 26° 44′ 30″ N and 108° 21′ 16″ E. The test site is located in the mid-subtropical monsoon climate zone, with an average annual temperature of 16.7 °C, an average annual rainfall of 1226 mm, and an average elevation of 820 m. The soil type is yellow loam, and the raw soil pH, total nitrogen, total phosphorus, total potassium, alkali-hydrolyzable nitrogen, soil-available phosphorus, soil-available potassium, and soil organic matter are 4.85, 1.33 g kg^−1^, 0.43 g kg^−1^, 10.02 g kg^−1^, 154 mg kg^−1^, 45.8 mg kg^−1^, 85 mg kg^−1^ and 30.95 g kg^−1^ respectively.

The six treatments utilized in this study were designated as follows: M1 (no soil conditioner), M2 (biomass ash), M3 (water retention agent), M4 (biochar), M5 (lime powder), and M6 (malic acid). On October 20th, 2020, green manure seeds (*Vicia sativa* L) were sown in all plots of the experimental field, and all six treatments were applied with chemical fertilizers and green manure. Experimental chemical fertilizers included urea (N 46%), calcium superphosphate (P_2_O_5_ 12%), and potassium sulfate (K_2_O 50%). Green manure (*Vicia sativa* L*)* was uniformly administered in all plots turned in situ and pressed into the soil at the base of the *Uncaria rhynchophylla* according to their growth. Each treatment consisted of an area of 15 m^2^ (3 m × 5 m) and was randomly replicated three times, resulting in a total of eighteen treatments on a 30° slope. There were 5 *Uncaria rhynchophylla* in each experimental plot, and the plots were spaced 0.5 m apart to the ridge to prevent water and fertilizer loss, and the side slopes of the experimental base were left more than 2 m for the protection rows. A basal fertilizer consisting of green manure, 60% urea, calcium superphosphate, and potassium sulfate was applied on April 5, 2021. A top-up fertilizer consisting of 40% urea was then applied on June 25, 2021. Biomass ash is a type of ash produced by burning local dead wood and fallen leaves; lime powder, biochar, water retention agent, and malic acid were purchased from the market. Further insights into specific soil conditioner and fertilizer compositions and their applications can be obtained from Text S1 and Table S1 in the Supplementary Material.

### Sample collection

Soil Sample Collection. Plant residues were removed before sampling and soil samples were collected according to the five-point sampling method. Each area was randomly drilled at five locations of 5 cm in diameter and 20 cm in depth using an open probe. Five soil samples were crushed and thoroughly mixed to form a composite soil sample, removing stones, branches, and plant roots to prepare 18 mixed soil samples (6 treatments × 3 replicates). Subsequently, 18 soil samples were passed through a 2 mm sieve, and part of the soil samples were naturally air-dried for the determination of soil physical and chemical properties, and part of the soil was stored at 4 °C for the determination of soil enzyme activities. The remaining soil samples were stored at -80 °C for microbial sequencing analysis.

Plant Sample Collection. Plant sampling was carried out on November 13, 2021, and three uniformly growing *Uncaria rhynchophylla* plants were randomly selected from each plot to test for yield and quality, and the samples collected were used for testing and analysis of related alkaloids in *Uncaria rhynchophylla*.

### Sample determination

Detailed procedures for the determination of soil physicochemical properties, including pH, total nitrogen (TN), total phosphorus (TP), total potassium (TK), alkali-hydrolyzable nitrogen (AHN), soil-available phosphorus (AP), soil-available potassium (AK), and soil organic matter (SOM) are described in our previous study^15^. In addition, methods for the determination of soil urease, phosphatase, sucrase, and catalase activities are presented in relevant references^16–18^. Total alkaloids were determined by HPLC-UV^19^. Isorhynchophylline, corynoxeine, and isocorynoxeine of *Uncaria rhynchophylla* were determined by the QuEChERS method as described in the Supplementary text S2.

### DNA extraction and metagenomic sequencing

To isolate DNA from each soil sample, 0.50 g of soil was processed using the FastDNA Spin Kit for Soil (MP Biomedicals, Santa Ana, CA, USA) according to the manufacturer's instructions. The sequencing was carried out at Shanghai Majorbio Biotechnology Co., Ltd. (Shanghai, China). Raw sequencing data underwent quality control using Fast v0.20.0 software to eliminate adapters and low-quality reads. Quality control data were then assembled using MEGAHI v1.1.2 software, requiring a minimum contig length of 300 bp. Following this, the assembled sequences were analyzed for open reading frames, with a minimum gene sequence length set at 100 bp. A mix-and-match strategy was employed for the assembly process to ensure comprehensive coverage. CD-HIT (http://www.bioinformatics.org/cdhit/) was utilized for all predicted genes, resulting in a non-redundant gene catalog. Gene abundance quantification was facilitated by the use of SOA Paligner (http://soap.genomics.org.cn/). Categorical annotations were conducted using the Basic Local Alignment Search Tool for Proteins (BLASTP) to compare the non-redundant gene catalog with the NCBI-NR database, using a value cutoff of 1e−523^20^.

### Statistical analysis

Data processing for soil physicochemical properties, enzyme activities, plant yield, and quality was carried out using Microsoft Excel 2016. For data analysis, we used IBM SPSS version 24.0 software for one-way analysis of variance (ANOVA). The results of the analysis were presented visually using Origin Pro 2022 software. In addition, the majorbio cloud platform was used for microbial community composition analysis of the soil samples. Principal coordinate analysis (PCoA) was performed using the Bray–Curtis distance algorithm to analyze changes in microbial community structure. Dominant correlation heatmaps were generated using RStudio (version 4.0.3).

### Plant guideline statement

The plants in this experiment were artificially planted and the collection, transport, and management of plant samples in the experiment were by national and international guidelines and legislation. The plant material used in this experimental design (*Uncaria rhynchophylla*) is not an endangered species listed by CITES. According to the International Union for Conservation of Nature (IUCN) Red List of Threatened Species, *Uncaria rhynchophylla* is classified as Least Concern (LC).

## Results and discussions

### Soil conditioner changes soil physicochemical properties

The results of a large number of current studies show that soil physicochemical properties, such as pH, bulk density, and soil organic carbon, are improved by the addition of soil amendments^21,22^. In this experiment, different soil conditioners had different effects on different soil properties^23^. Low soil pH results in soil acidification, which represents a significant challenge for modern Chinese agriculture^24^. The results demonstrated that five soil conditioners could improve soil pH and alleviate soil acidification. Soil pH increased by 1.49–13.62% in the additional application of various soil conditioner treatments compared to M1 (*p* < 0.05; Table 1). The most significant effect was observed with lime powder, which is associated with the alkaline nature of lime powder^25^. AHN or TN levels are commonly used indicators for assessing soil N supply capacity^26^. The M2 treatment exhibited the sole increase in AHN content, with the maximum values observed for soil TN and SOM (*p* < 0.05; Table 1). It has been shown that the application of biomass ash soil conditioners to agricultural activities can improve the soil environment and increase the nutrient content^27^. In addition, there was no significant difference in soil TP, AP, and TK after soil conditioner application compared to M1 treatment (*p* > 0.05; Table 1). From the results of this study, M2 can increase soil pH, TN, TP, TK, AHN, AP, AK, and SOM, and compared with other soil conditioners, M2 has a better effect on the comprehensive improvement of *Uncaria rhynchophylla* soil.

**Table 1:** Soil properties under the treatment of different soil conditioners.

Treatment	PH	TN	TP	TK	AHN	AP	AK	SOM
		g·kg^−1^	g·kg^−1^	g·kg^−1^	mg·kg^−1^	mg·kg^−1^	mg·kg^−1^	g·kg^−1^
M1	4.70 ± 0.10b	1.44 ± 0.35a	0.39 ± 0.12a	8.53 ± 1.07a	145.67 ± 14.19ab	34.33 ± 7.13ab	101.50 ± 16.26c	34.77 ± 9.00a
M2	4.84 ± 0.09b	1.60 ± 0.17a	0.45 ± 0.05a	8.87 ± 1.54a	149.67 ± 8.02a	35.73 ± 1.19ab	125.50 ± 13.44bc	38.99 ± 3.62a
M3	4.86 ± 0.20b	1.32 ± 0.45a	0.44 ± 0.25a	9.47 ± 2.02a	131.89 ± 18.33abc	50.00 ± 16.02a	135.50 ± 14.85b	37.24 ± 4.19a
M4	4.79 ± 0.10b	1.55 ± 0.31a	0.45 ± 0.07a	9.27 ± 2.23a	144.00 ± 13.75ab	32.33 ± 3.93b	116.50 ± 6.36bc	38.40 ± 8.60a
M5	5.34 ± 0.22a	1.46 ± 0.22a	0.48 ± 0.08a	9.57 ± 2.38a	111.00 ± 15.88c	41.00 ± 9.97ab	188.00 ± 15.56a	36.68 ± 7.14a
M6	4.77 ± 0.18b	1.41 ± 0.37a	0.39 ± 0.11a	9.73 ± 2.26a	121.00 ± 39.15bc	34.53 ± 6.59ab	69.50 ± 2.12d	32.99 ± 9.53a

M1, No soil conditioner; M2, Biomass ash; M3, Water retention agent; M4, Biochar; M5, lime powder; M6, Malic acid; TN, Total nitrogen; TP, Total phosphorus; TK, Total potassium; AHN, Alkali-hydrolyzable nitrogen; AP, Soil-available phosphorus; AK, Soil-available potassium; SOM, Soil organic matter. Data are shown as mean with standard deviation. Different letters within a column indicate significant differences at *p* < 0.05.

### Soil conditioner changes the composition, diversity, and community structure of soil microbial communities

The Chao 1, ACE, Shannon, and Simpson indices were employed to quantify microbial species abundance and diversity to identify the effects of different soil conditioners on soil microbial community composition (Fig. S1). All groups of bacterial community abundance and diversity indices exhibited varying degrees of statistical significance. In our study, the alpha diversity indices ACE, Chao 1, and Shannon decreased and Simpson increased in the soil conditioner application treatments compared to M1 (Fig. S1). The results of this study indicated that the application of soil conditioners led to a decrease in microbial abundance and diversity. It may be because the application of soil amendments promotes the colonization of certain microorganisms in the soil but results in reduced diversity due to insufficient competitiveness of other microbiota^28^.

**Figure 1:**
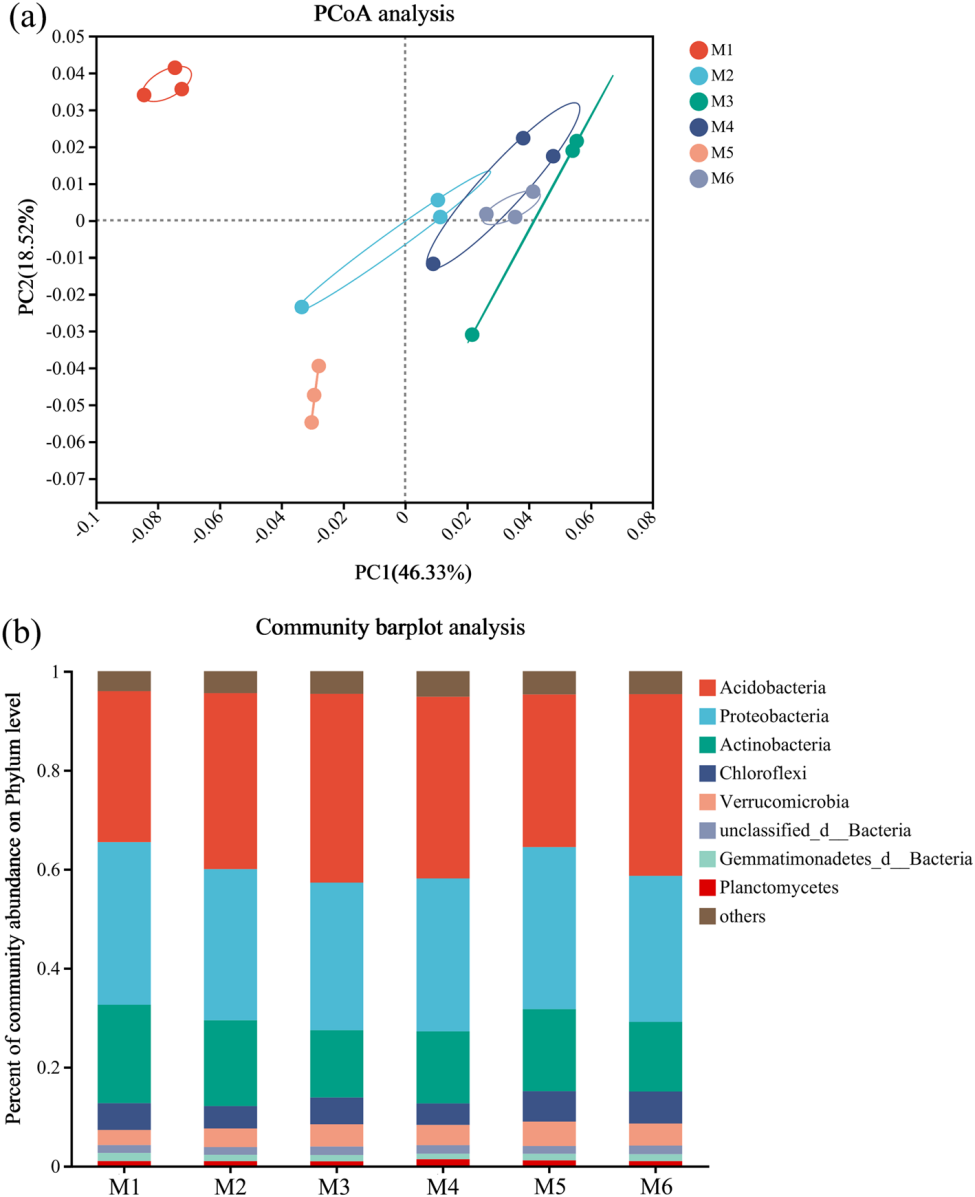
Principal component analysis (**a**) and bacterial community abundance (**b**) on phylum level during the different treatments.

Beta diversity was evaluated using principal coordinate analysis to analyze differences in species community composition and structure. The first axis accounted for 46.33% of the correlation in bacterial community structure, while the second axis accounted for 18.52%, collectively explaining 64.85% of the total variance (Fig. 1a). It was found that the community structure was similar between the soil conditioner application treatments, but there were some differences in community structure compared to M1. Therefore, soil conditioner treatments affected the structure of soil bacterial communities to some extent, and promoted more diverse microbial communities^14^.

**Figure 2:**
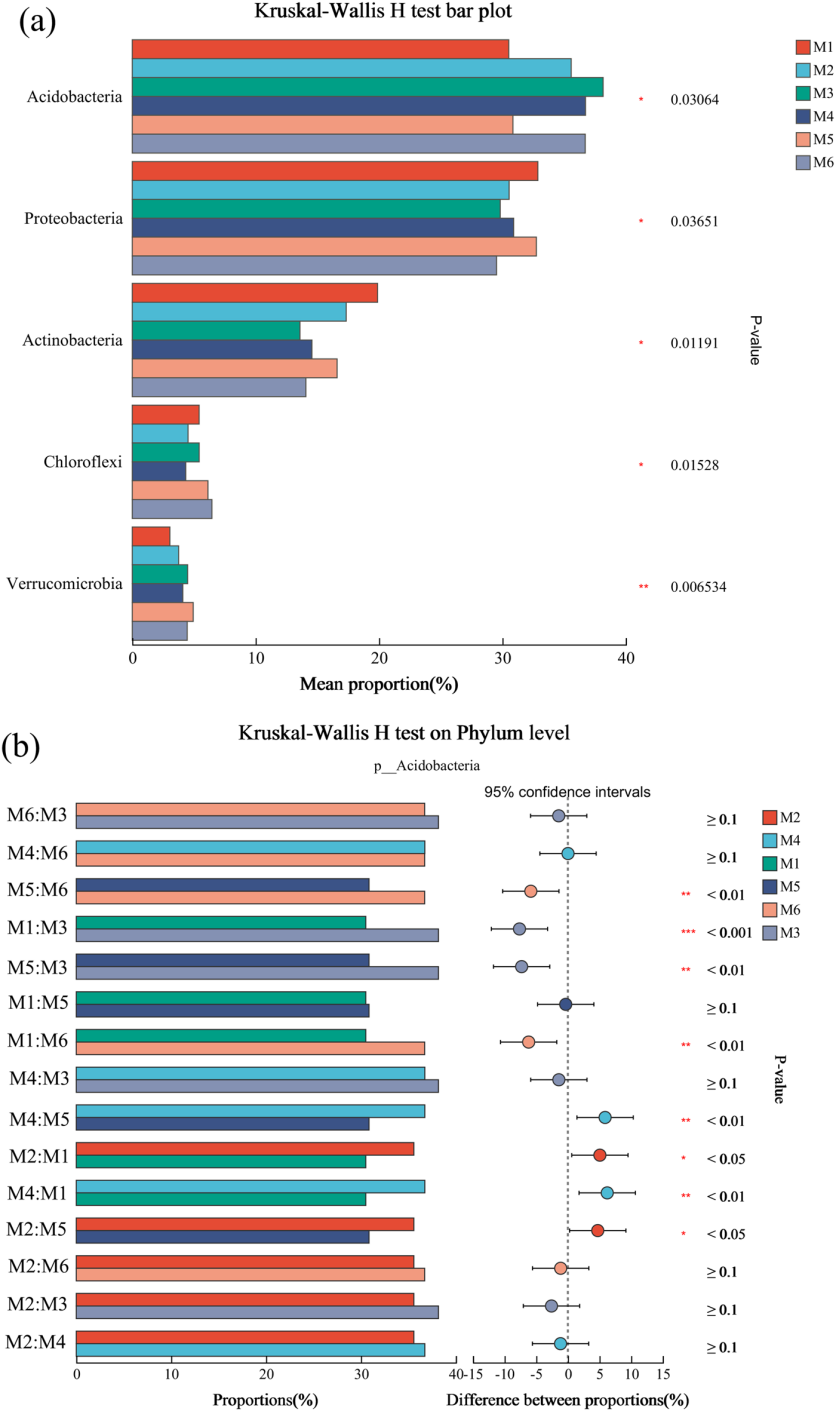
Relative abundances (**a**) and bar plot on phylum level (**b**) of bacterial phyla in the *Uncaria rhynchophylla* soil.

Soil microorganisms represent integral components of terrestrial ecosystems, with their diversity and community composition serving as vital indicators of biotransformation efficiency and soil fertility status^29,30^. The taxonomic distribution and relative abundance of the major clades are shown in Fig. 1b and Table S2. The dominant phyla in all soil samples were *Acidobacteria* (30.51–38.15%), *Proteobacteria* (29.52–32.87%), *Actinobacteria* (13.59–19.88%), and *Chloroflexi* (4.34–6.46%), which together accounted for 77.96–97.36% of the bacterial sequences. The *Acidobacteria* phylum was the dominant phylum with the highest relative abundance, and the relative abundance of the *Acidobacteria* phylum differed among treatments (Fig. 2a;* p* < 0.05). Further analysis of the test of variance for the *Acidobacteria* phylum revealed that the relative abundance of *Acidobacteria* was significantly higher in each soil conditioner treatment than in the M1 treatment, except for M5 (Fig. 2b;* p* < 0.05). *Acidobacteria* has been associated with the promotion of indole-3-acetic acid and iron carriers in plants, resulting in significant enhancement of plant growth parameters^31^. Additionally, *Acidobacteria* have been linked to SOM decomposition, facilitating soil SOM decomposition and denitrification, thus improving soil carbon stability^32^. In contrast to the changes in *Acidobacteria*, the relative abundance of *Actinobacteria* and *Chloroflexi* was significantly reduced by the application of soil conditioners M2 and M4 (Fig. 2a;* p* < 0.05). This decrease may be due to the alteration of the soil environment by biochar, such as the ability to fix CO_2_ and the inhibition of nitrification and ammonia oxidation processes, which indirectly affects the survival environment of *Actinobacteria* and *Chloroflexi*^33^. In addition, the application of soil conditioners resulted in a significant increase in the relative abundance of *Verrucomicrobia* (Fig. 2a;* p* < 0.05). *Verrucomicrobia* is a beneficial soil bacterium that is mainly involved in the decomposition of organic matter, and nutrient cycling in the soil, and promotes plant growth^34^. In summary, the application of soil conditioners can alter the soil microbial diversity and microbial community structure.

### Soil conditioner changes the soil enzyme activity

**Figure 3:**
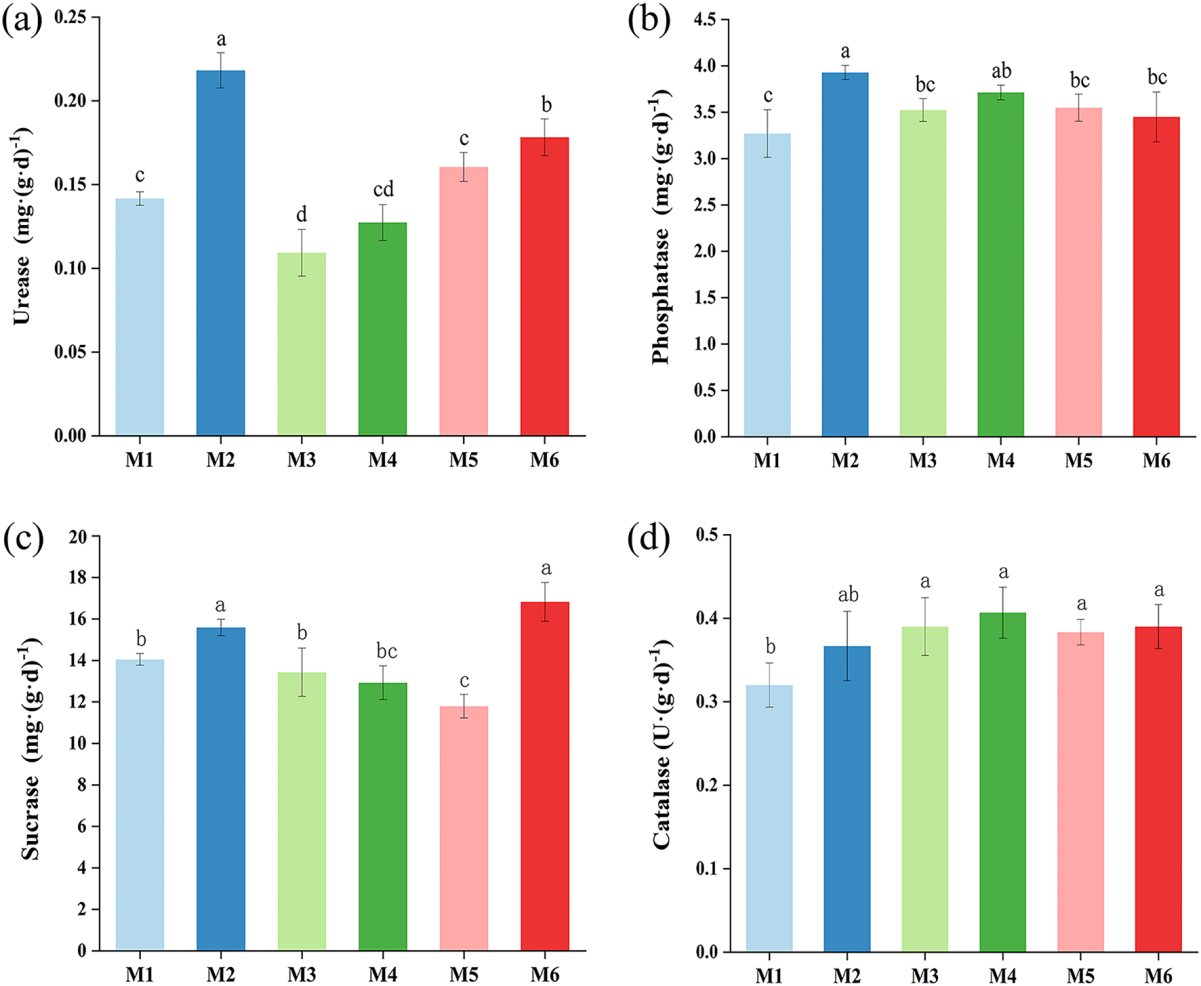
Soil enzyme activity at the *Uncaria rhynchophylla* harvesting stage. (**a**) Urease activity; (**b**) Phosphatase activity; (**c**) Sucrase activity; (**d**) Catalase activity. Different letters within a column indicate significant differences at *p* < 0.05.

Soil enzymes, primarily urease, catalase, phosphatase, and sucrase, play a crucial role in soil biochemical processes. They serve as crucial indicators of soil fertility, pollution, and environmental change^35^. It showed that increased application of soil conditioner increased soil phosphatase and catalase activities compared to M1, where soil phosphatase activities were significantly increased by 20.16% and 13.55% in M2 and M4, respectively (*p* < 0.05; Fig. 3b). Furthermore, other studies have demonstrated that soil conditioners can increase soil phosphatase activity^36^. In addition, soil catalase activity was significantly increased by 15.63–28.13% in M2-M6 compared to M1. Among them, M4 increased soil catalase activity by 28.13% (*p* < 0.05; Fig. 3d). Decreased soil catalase activity had a detrimental effect on soil ecosystem homeostasis^37^, which highlights the ability of soil conditioners to enhance soil ecosystems and maintain ecological balance. Urease and sucrase displayed distinct trends across the various treatments. Biomass ash (M2) and malic acid (M6) increased soil urease activity compared to M1 without soil conditioner, where biomass ash (M2) treatment significantly soil urease increased by 53.52% (Fig. 3a). It is worth noting that the application of soil conditioners can improve soil nutrient content and increase soil enzyme activity^38^. Therefore, the increase in urease activity after the application of biomass ash soil conditioners may further improve the soil environment by facilitating nutrient cycling^39^. Application of biomass ash (M2) and malic acid (M6) treated soil sucrase activity significantly increased by 10.95% and 19.71%, respectively, compared to M1 without soil conditioner (Fig. 3c). It is known that sucrase activity is related to SOM and microbial population and its activity is positively correlated with soil fertility^40^, suggesting that M2 and M6 have positive effects on soil fertility improvement. In summary, the application of green manure with biomass ash could be effective in increasing the soil enzyme activities of *Uncaria rhynchophylla*.

### Soil conditioner changes *Uncaria rhynchophylla* yield and quality

As shown in Table 2, soil conditioner application altered the yield of *Uncaria rhynchophylla*. Fresh weight increased significantly (*p* < 0.05) by 16.46%, 17.72%, and 25.32% in M2, M3, and M4, respectively, as compared to control treatment M1. The variation in dry weight was similar to that in fresh weight with a significant difference of 25.00% increase in the M2 treatment. It may be because biomass ash itself is rich in nutrients such as potassium, calcium, and magnesium for plant growth^41^, which promotes the growth of *Uncaria rhynchophylla* and corresponds to the changes in soil nutrients in Table 1. It is worth noting that the application of biochar conditioner has previously been reported to promote plant growth and increase crop yields^42^, which is consistent with the results of our study. In addition, the application of the lime powder conditioner M5 did not promote the increase in the increase of *Uncaria rhynchophylla* yield, while the results of other studies also showed that the application of the lime powder conditioner reduced the yield of cabbage^43^.

**Table 2:** Alkaloid content and yield of *Uncaria rhynchophylla* with different soil conditioners.

Treatment	Isorhynchophylline (mg·kg^−1^)	Corynoxeine (mg·kg^−1^)	Isocorynoxeine (mg·kg^−1^)	Total alkaloid (mg·kg^−1^)	Fresh weight (mg·g^−1^)	Dry weight (mg·g^−1^)
M1	25.61 ± 1.36b	22.55 ± 0.86c	11.12 ± 0.27d	2.26 ± 0.19bc	0.79 ± 0.03b	0.36 ± 0.03b
M2	32.00 ± 1.54a	32.14 ± 1.41a	17.82 ± 0.41a	3.14 ± 0.10a	0.92 ± 0.03a	0.45 ± 0.02a
M3	12.60 ± 0.38d	9.08 ± 0.40e	5.73 ± 0.73e	2.04 ± 0.12c	0.93 ± 0.05a	0.43 ± 0.04ab
M4	27.85 ± 2.27b	30.71 ± 1.97ab	16.18 ± 0.85b	2.34 ± 0.43bc	0.99 ± 0.01a	0.41 ± 0.04ab
M5	18.91 ± 1.40c	18.17 ± 1.43d	7.56 ± 0.56e	2.17 ± 0.07c	0.60 ± 0.08c	0.28 ± 0.04c
M6	33.47 ± 2.24a	28.73 ± 2.55b	14.52 ± 0.15c	2.65 ± 0.16b	0.79 ± 0.04b	0.38 ± 0.04ab

Alkaloids are the main active ingredients of *Uncaria rhynchophylla*. The content of isorhynchophylline, corynoxeine, isocorynoxeine, and total alkaloids in *Uncaria rhynchophylla* were determined (Table 2). The application of soil conditioners M2, M4, and M6 resulted in an increase in isorhynchophylline, corynoxeine, and isocorynoxeine compared to M1, with the highest alkaloid content in the M2 treatment. Specifically, the content of these isorhynchophylline, corynoxeine, and isocorynoxeine was elevated by 24.95%, 42.53%, and 60.25%, respectively. Similarly, the effect of soil conditioners on total alkaloid content showed a similar trend with a significant increase of 38.94% in M2 treatment. The results showed that the content of isorhynchophylline, corynoxeine, isocorynoxeine, and total alkaloids in the M2 treatment was superior to the other treatments, which could be attributed to the fact that the biomass ash improved the soil properties and increased the soil organic matter, which in turn benefited the growth and development of the plant, and consequently, improved the yield quality of *Uncaria rhynchophylla*^27^.

### Relationship between yield and quality of *Uncaria rhynchophylla*, soil properties, microbial communities, and enzyme activities

**Figure 4:**
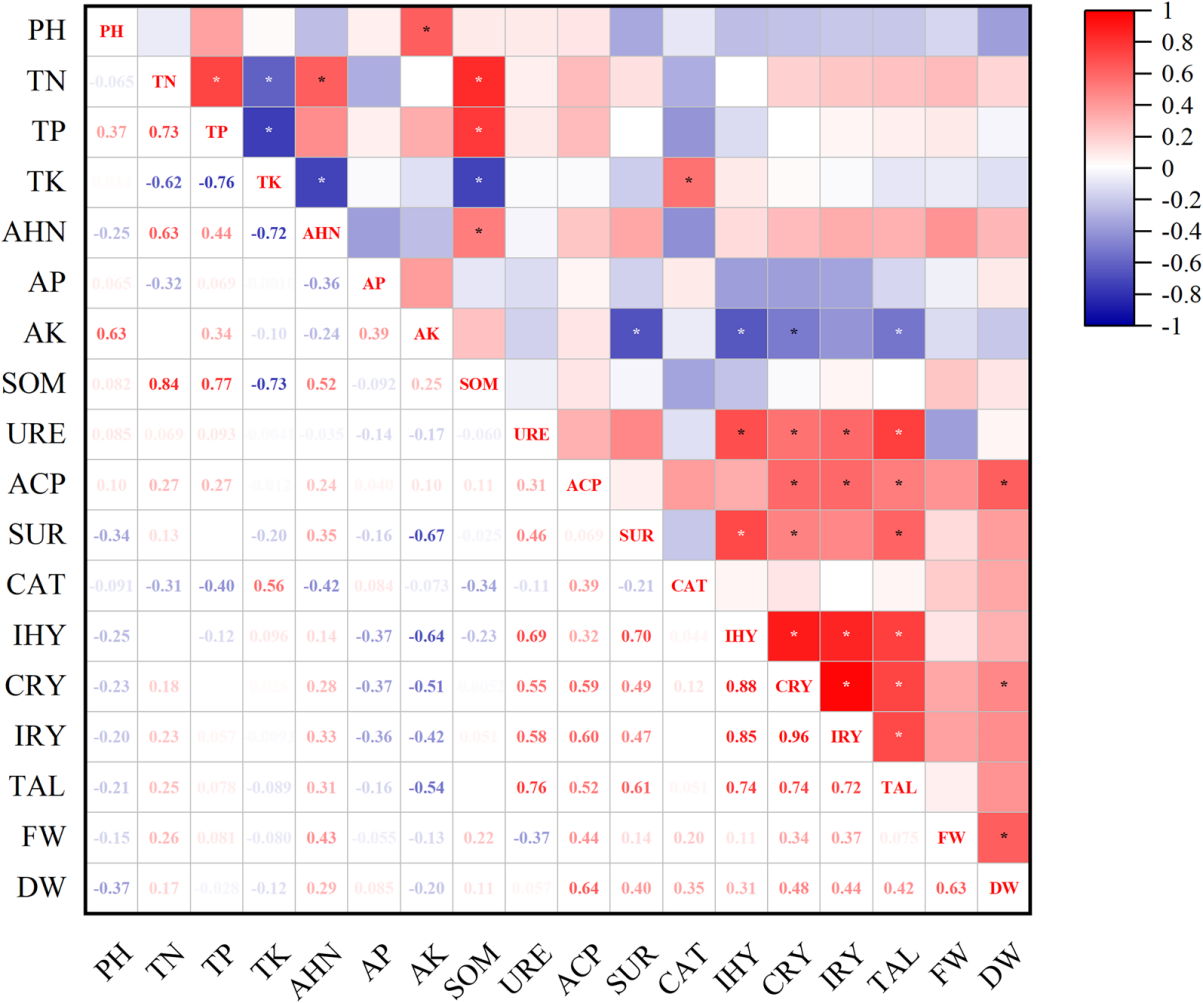
Spearman’s correlation analysis (*p*, 0.05 *) is used to study the correlation between yield, quality, soil physicochemical properties, and enzyme activity of *Uncaria rhynchophylla*. TN, Total nitrogen; TP, Total phosphorus; TK, Total potassium; AHN, Alkali-hydrolyzable nitrogen; AP, Soil-available phosphorus; AK, Soil-available potassium. SOM, Soil organic matter; URE, Urease; ACP, Phosphatase; SUR, Sucrase; CAT, Catalase; IHY, Isorhynchophylline; CRY, Corynoxeine; IRY, Isocorynoxeine; TAL, Total alkaloid; FW, Fresh weight of hooked branches; DW, Dry weight of hooked branches. “*” indicates *p* < 0.05 significant correlation, respectively.

The correlation analysis between soil properties, soil enzyme activities, *Uncaria rhynchophylla* alkaloid content, and yield is shown in Fig. 4. The results showed that dry weight was significantly and positively correlated with phosphatase activity (*p* < 0.05), corynoxeine, isocorynoxeine, and total alkaloid content were significantly and positively correlated with urease and phosphatase (*p* < 0.05). This indicates that phosphatase activity is an important yield and quality factor in *Uncaria rhynchophylla*. As shown in Fig. 3b, the application of soil conditioners can increase soil phosphatase activity, which in turn explains the positive effect of soil conditioners in improving crop yield and quality^44,45^. Application of soil conditioners showed different trends for urease activity. Among them, soil conditioners increased urease activity except M3 and M4, with M2 promoting urease activity the most effectively (Fig. 3a). Similarly, we also observed in Table 2 that corynoxeine, isocorynoxeine and total alkaloid content of *Uncaria rhynchophylla* were lower in M3 than in M1 with some significance, but M4 showed a decreasing trend, which may be affected by other unknown environmental disturbances. Overall, the application of M2 soil conditioner increased the urease and phosphatase activities of *Uncaria rhynchophylla*, which may be one of the reasons why M2 improved the yield and quality of *Uncaria rhynchophylla*.

**Figure 5:**
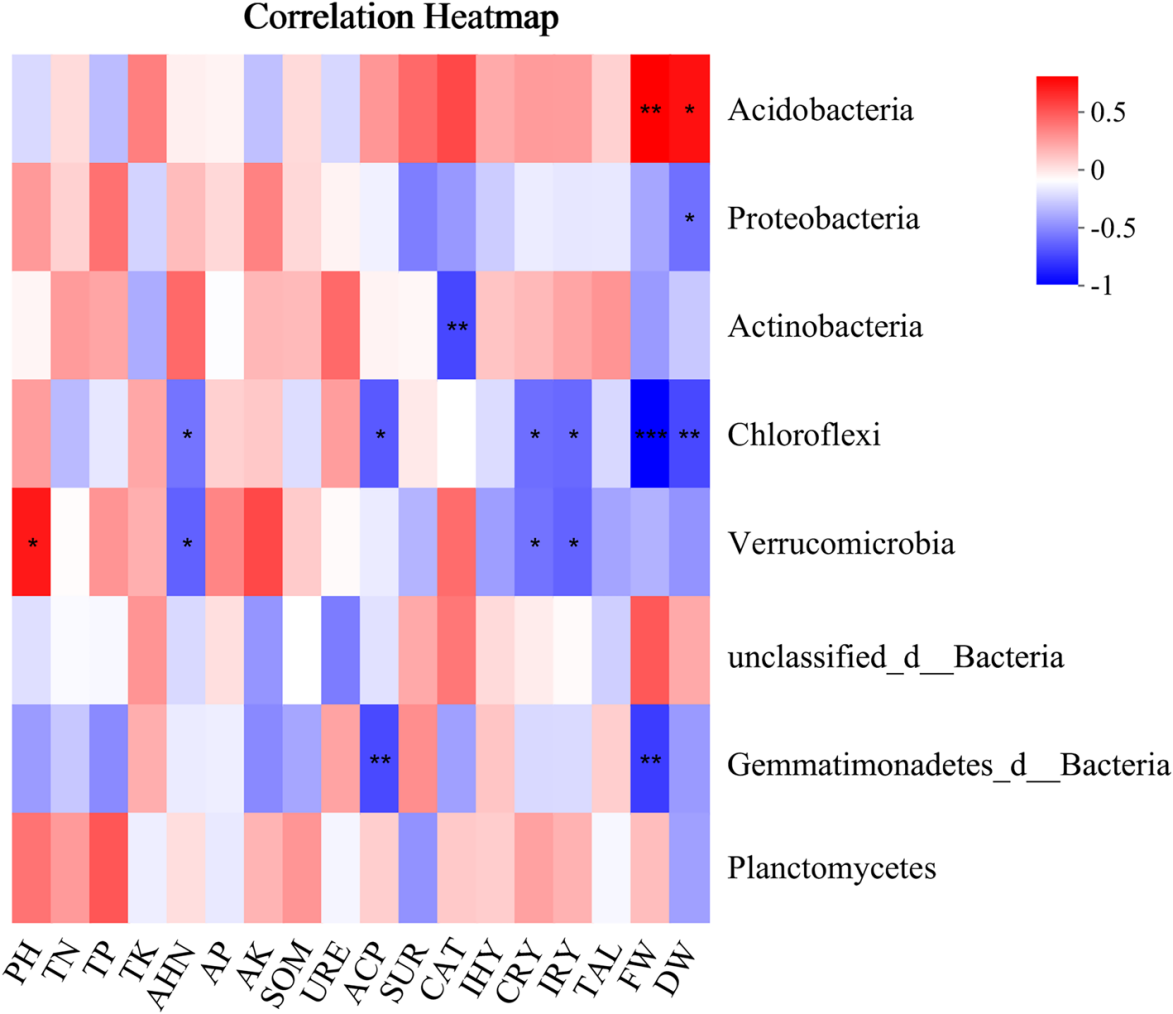
Correlation heatmap analysis between environmental factors and bacterial species by Bray–Curtis distance based on phylum level. TN, Total nitrogen; TP, Total phosphorus; TK, Total potassium; AHN, Alkali-hydrolyzable nitrogen; AP, Soil-available phosphorus; AK, Soil-available potassium; SOM, Soil organic matter; URE, Urease; ACP, Phosphatase; SUR, Sucrase; CAT, Catalase; IHY, Isorhynchophylline; CRY, Corynoxeine; IRY, Isocorynoxeine; TAL, Total alkaloid; FW, Fresh weight; DW, Dry weight. “*” “**” “***” indicates *p* < 0.05, *p* < 0.01 and *p* < 0.001 significant correlation, respectively.

The correlation analysis between soil properties, soil enzyme activities, *Uncaria rhynchophylla* alkaloid content and yield, and soil microbial community structure is shown in Fig. 5. The relative abundance (top five) of microbial communities in this experiment was not only closely related to soil physicochemical properties and soil enzyme activities but also influenced the yield and quality of *Uncaria rhynchophylla*. The relative abundance of *Acidobacteria*, *Proteobacteria*, *Actinobacteria*, *Chloroflexi,* and *Verrucomicrobia* was significantly correlated with pH, AHN, ACP, CAT, CRY, IRY, FW, and DW (Fig. 5). In our study, the relative abundance of *Acidobacteria* was significantly positively correlated with fresh and dry weight. Application of soil conditioners increased the abundance of *Acidobacteria* (except for M5), suggesting that application of soil conditioning may contribute to the enhancement of *Uncaria rhynchophylla* yield (Fig. 2b). The relative abundance of *Chloroflexi* was significantly negatively correlated with alkali-hydrolyzable nitrogen, phosphatase activity, corynoxeine, and isocorynoxeine (*p* < 0.05; Fig. 5), and highly significantly negatively correlated with fresh and dry weight (*p* < 0.01; Fig. 5). Phosphatase activity was positively correlated with isorhynchophylline, corynoxeine, and isocorynoxeine, total alkaloids, and dry weight (*p* < 0.05; Fig. 4). This may be related to the fact that *Chloroflexi* belongs to the negative nutrient group of bacteria, whose relative abundance decreases with the application of fertilizers^46^ as well as to the fact that soil amendments can increase phosphatase activity and promote soil organophosphorus mineralization^47^. *Verrucomicrobia* are common microorganisms in soil, where their members are involved in the decomposition of organic matter, nutrient cycling, and interactions with other microorganisms to promote plant growth^34^. However, contrary to our findings, the relative abundance of *Verrucomicrobia* was significantly (*p* < 0.05) negatively correlated with alkali-hydrolyzable nitrogen, corynoxeine, and isocorynoxeine. The effects of *Verrucomicrobia* on *Uncaria rhynchophylla* plant quality were shown to be both promoting and inhibiting, which deserves to be followed up and further investigated. However, soil microbial activity is a complex process that can be influenced by many factors.

In summary, soil conditioner M2 can improve *Uncaria rhynchophylla* yield and quality by improving soil properties, enzyme activities, and soil microbial community structure, such as increasing soil alkali-hydrolyzable nitrogen, increasing urease and phosphatase activities, increasing the abundance of the *Acidobacteria* and decreasing the abundance of the *Chloroflexi*.

## Conclusions

This study evaluated the potential benefits of soil conditioners in the cultivation of *Uncaria rhynchophylla* by comparing the effects of different soil conditioners on the properties, soil microbial diversity and community structure, and soil enzyme activities of *Uncaria rhynchophylla* soils in Guizhou, with special attention to crop yield and quality. The results of the study showed that the increased application of soil conditioner alleviated soil acidification compared to the application of green manure and chemical fertilizer alone. In addition, it increased the activities of urease, phosphatase, catalase, and sucrase and caused significant changes in the composition of soil microbial communities. Correlation analysis showed that *Acidobacteria* was significantly and positively correlated with the fresh and dry weight of *Uncaria rhynchophylla*, while *Chloroflexi* was significantly and negatively correlated with alkali-hydrolyzable nitrogen, phosphatase activity, fresh and dry weight, corynoxeine, and isocorynoxeine. Specifically, the application of soil conditioner M2 increased the abundance of *Acidobacterium* and decreased the abundance of *Chloroflexi*, which increased the soil nutrient content and improved the yield and quality of *Uncaria rhynchophylla*. As a green and energy-saving material prepared from agricultural wastes (dead wood and fallen leaves), M2 is economical, environmentally friendly, and highly efficient compared with other soil conditioners. In summary, M2 treatment is an ideal soil conditioner for regulating the structure of the microbial community, promoting the yield and quality of *Uncaria rhynchophylla*. Therefore, M2 is recommended to be selected as a soil conditioner to promote the cultivation area of *Uncaria rhynchophylla*.

### Supplementary Information

Supplementary Information.

## Data Availability

The data from this study was deposited in the NCBI Sequence Read Archive under accession SRA: SRP498352.
