# Supplementary material for: Soil conditioner improves soil properties, regulates microbial communities, and increases yield and quality of Uncaria rhynchophylla
Source: Sci Rep. 2024 Jun 11;14:13398. doi: 10.1038/s41598-024-64362-4 (PMC11167052; doi:10.1038/s41598-024-64362-4)
Supplement: Supplementary file 1 — Supplementary Information. [file 41598_2024_64362_MOESM1_ESM.pdf]

Supplementary Material

Soil conditioner improves soil properties, regulates microbial communities, and increases yields and quality of *Uncaria rhynchophylla*

Qian Liu[1], Honghao Cui[1,2], Wansheng Yang[1], Fang Wang[3], Heng Liao[2], Qing Zhu[2], Song Qin[2*] & Ping Lu[1*]

[1]Center for R&D of Fine Chemicals, Guizhou University, Guiyang 550025, China. [2]Institute of Soil Fertilizer, Guizhou Academy of Agricultural Sciences, Guiyang 550006, China. [3]Guizhou Industry Polytechnic College, Guiyang 550008, China.

Corresponding Author:

Email: aas_qinsong@126.com; plu@gzu.edu.cn.

Text S1. The composition information of conditioners.

Biomass ash (M2) is a homemade grass ash composed of ash powder left after collecting local dead wood, fallen leaves, etc., burned. Its main elements are 3.65% potassium, 3.4% calcium, 2.1% silicon, and 1% magnesium. The principal component of the water-retaining agent (M3) is a high-water-absorption resin, procured from Henan Bafus Chemical Products Co., Ltd. Biochar (M4) was procured from Henan Lize Environmental Protection Technology Co., Ltd. with a carbon content of 420 g/kg. The main components were as follows: organic carbon (42.21 g/kg), total nitrogen (8.34 g/kg), total phosphorus (2.31 g/kg), total potassium (16.12 g/kg), pH (9.46), and 7.23 g/kg of ash powder. Lime powder (M5) was primarily composed of calcium oxide, containing approximately 95% calcium. It was procured from the Fenyi Huangbaozhu Daily Chemical Products Co., Ltd. Malic acid (M5) was purchased from Anhui Xuelang Bio-technology Co., Ltd. The main component is DL-malic acid.

Text S2. Detailed information about the isorhynchophylline, corynoxeine, and isocorynoxeine.

1. Preparation methods

Weighed 0.3 g of powder from the hooked parts of UR, added 30 mL of extractant, soaked for 12 h, extracted by ultrasonication for 30 min, centrifuged, passed through a 0.22 μm organic membrane, and detected by LC-MS/MS.

2. LC-MS/MS condition

Isorhynchophylline, corynoxeine, and isocorynoxeine were separated by the Shimadzu 20 AD-XR liquid chromatography system (Tokyo, Japan) using a Phenomenex Kinetex F5 (100 × 1.7 mm, 2.5 μm) with columns temperature of 35°C.

The flow rate of mobile phases was 0.32 mL min^{-1} and the injection volume was1 μL. The mobile phase was acetonitrile (A) and 0.1% formic acid in water (B). Results were confirmed using an AB Sciex 4500Q trap mass spectrometer device (Foster City, CA, USA). Multiple reaction monitoring (MRM) modes and a positive mode electrospray ionization source (ESI+) were used to determine concentrations of isorhynchophylline, corynoxeine, and isocorynoxeine. The ESI parameters are as follows: ion spray voltage, 5500 V; ion source temperature, 600°C; curtain gas 30 psi, as for imidacloprid ion source gases 1 and 2 pressures were 60 psi. As for isorhynchophylline, the transitions employed for qualification and qualitative analysis were 385.3/241.0 and 385.3/353.2, and collision energies were 44.52 and 23.72 eV, respectively. As for corynoxeine, the transitions employed for qualification and qualitative analysis were 383.2/267.1 and 383.2/351.2, and collision energies were 28.36 and 24.18 eV, respectively. As for isocorynoxeine, the transitions employed for qualification and qualitative analysis were 383.0/201.0 and 383.0/351.1, and collision energies were 30.78 and 25.21 eV, respectively.

Table S1. Fertilization amount of soil conditioner.

Treatment	Biomass ash	Water retention agent	Biochar	Lime powder	Malic acid	N1	N2	P_2O_5	K_2O	Green manure
	(kg $(15m^2)^{-1}$)									
M1	0	0	0	0	0	0.36	0.24	0.1	0.258	34
M2	1.125	0	0	0	0	0.36	0.24	0.1	0.258	34
M3	0	0.045	0	0	0	0.36	0.24	0.1	0.258	34
M4	0	0	1.125	0	0	0.36	0.24	0.1	0.258	34
M5	0	0	0	1.125	0	0.36	0.24	0.1	0.258	34
M6	0	0	0	0	0.01125	0.36	0.24	0.1	0.258	34

Note: M1 (no soil conditioner), M2 (biomass ash), M3 (water retention agent), M4 (biochar), M5 (lime powder), and M6 (malic acid); N1, First application of nitrogen fertilizer (base fertilizer); N2, Second application of nitrogen fertilizer (additional fertilizer). Green manure (*Vicia sativa* L) and chemical fertilizers are applied at the recommended rate using the principle of balanced fertilizer application.

Table S2. Raw data of relative abundance of bacterial phyla in *Uncaria rhynchophylla* soil.

Phylum	M1	M2	M3	M4	M5	M6
p__Acidobacteria	0.3051	0.3556	0.3815	0.3673	0.3085	0.3671
p__Proteobacteria	0.3287	0.3054	0.2983	0.3091	0.3274	0.2952
p__Actinobacteria	0.1988	0.1735	0.1359	0.1456	0.1660	0.1407
p__Chloroflexi	0.0542	0.0452	0.0542	0.0434	0.0613	0.0646
p__Verrucomicrobia	0.0305	0.0376	0.0449	0.0410	0.0494	0.0445
p__unclassified_d__Bacteria	0.0160	0.0156	0.0172	0.0172	0.0157	0.0174
p__Gemmatimonadetes_d__Bacteria	0.0163	0.0128	0.0127	0.0111	0.0133	0.0140
p__Planctomycetes	0.0101	0.0099	0.0096	0.0138	0.0115	0.0100
others	0.0404	0.0443	0.0457	0.0515	0.0470	0.0465

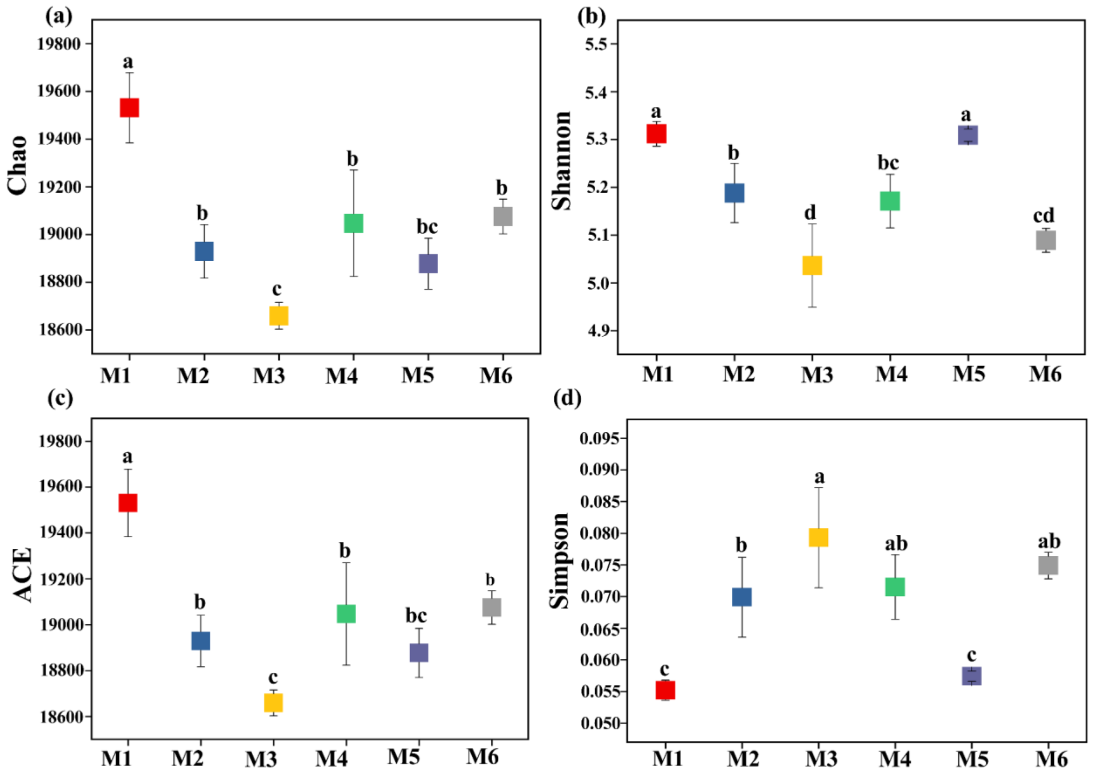

Fig. S1. Significant box chart of the α-diversity index of *Uncaria rhynchophylla* under different soil conditioners. (a) Chao; (b) Shannon; (c) ACE; (d) Simpson. Chao indicates Chao 1.
